# Single-Centre Case Series Assessment of Early Exercise Capacity Data Among Patients Who Received an Alterra Prestent and SAPIEN 3 Valve Placement

**DOI:** 10.1016/j.cjcpc.2022.06.002

**Published:** 2022-06-17

**Authors:** William B. Orr, Jamie N. Colombo, Bayley Roberts, Jennifer N. Avari Silva, David Balzer, Shabana Shahanavaz

**Affiliations:** a Division of Pediatric Cardiology, Department of Pediatrics, Washington University School of Medicine, St. Louis, Missouri, USA; b Department of Biomedical Engineering, Washington University McKelvey School of Engineering, St. Louis, Missouri, USA; c The Heart Institute, Cincinnati Children's Hospital Medical Center, Cincinnati, Ohio, USA

## Abstract

Previous studies have used cardiopulmonary exercise test (CPET) data to objectively assess physiological changes in patients undergoing percutaneous pulmonary valve implantation. A retrospective review was performed to assess pre- and post-CPET data among patients undergoing Alterra Adaptive Prestent and SAPIEN 3 transcatheter heart valve (Alterra) placement. Of the 7 patients eligible for the study, 5 (71%) were male. The mean age was 22 years (range: 12-49 years). CPET data showed significant (*P* = 0.03) improvement in ventilatory efficiency (V_E_/V_CO2_) while only 2 (29%) patients had an improvement of percent predicted peak oxygen consumption (V_O2_). These findings suggest favourable haemodynamic changes though further investigation is needed.

## Résumé

Des résultats aux épreuves d’effort cardiorespiratoire ont été utilisés lors d’études antérieures pour mesurer de manière objective les changements physiologiques chez les patients ayant subi l’implantation percutanée d’une valvule pulmonaire. Nous présentons une étude rétrospective des résultats à ces épreuves avant et après l’intervention dans des cas d’implantation transcathéther d’une prothèse Alterra Adaptive Prestent et d’une valve cardiaque SAPIEN 3 (Alterra). Parmi les sept patients admissibles à l’étude, cinq (71 %) étaient de sexe masculin. L’âge moyen des sujets était de 22 ans (plage de 12 à 49 ans). Les résultats obtenus à l’épreuve d’effort cardiorespiratoire ont démontré une amélioration significative (*P* = 0,03) de l’efficacité respiratoire (V_E_/V_CO2_), mais seulement deux patients (29 %) ont présenté une amélioration du pourcentage prévu de la consommation maximale d’oxygène (V_O2_). Bien que ces observations semblent indiquer des changements hémodynamiques favorables, d’autres études sont nécessaires pour élucider la question.

Changes in exercise capacity have been measured and reported after both surgical and percutaneous pulmonary valve implantations (PPVI).^1^, ^2^, ^3^, ^4^ However, the PPVI data have been limited to the Melody transcatheter pulmonary valve (Medtronic, Inc, Dublin, Ireland) and the Edwards SAPIEN 3 valve (Edwards Lifesciences, Irvine, CA). In 2012, a large investigational trial studying exercise function among patients who underwent a PPVI with the Melody valve showed modest improvement in exercise capacity and gas exchange efficiency.^1^ Until recently, there were no percutaneous options for patients who had right ventricular outflow tract (RVOT) too dilated to accommodate either a Melody or SAPIEN 3 valve leaving only surgical options.

An RVOT previously considered too large for PPVI may now have additional transcatheter options, including the Alterra Adaptive Prestent and SAPIEN 3 transcatheter heart valve (THV) system (Alterra). The Alterra system uses a docking adaptor for the 29-mm SAPIEN 3 THV within the RVOT as described in detail by Zahn et al.^5^

The primary aim of this study is to gain early insight into changes in exercise capacity among patients receiving percutaneous Alterra placement and to compare it with published data on other PPVI. We hypothesize that patients undergoing Alterra prestent/SAPIEN 3 valve placement would reflect similar benefits in exercise tolerance to the other patients undergoing PPVI.

## Materials and Methods

A retrospective chart review was performed from July 2019 until July 2021 using both adult and paediatric patients who had an Alterra placed and had a cardiopulmonary exercise test (CPET) at our institution. Patients were excluded if they did not have a baseline CPET and a 6-month post-Alterra placement CPET. Patients were also excluded if the peak respiratory exchange ratio (RER) was less than 1.05 or if the peak heart rate (HR) was less than 85% of predicted, which was used to indicate a submaximal effort. This study received approval from the Washington University School of Medicine institutional review board (IRB#: 202102132).

Patient demographic data including age, weight, body mass index, gender, original lesion, number of surgeries, time since the original repair, and Alterra prestent and SAPIEN 3 THV placement indication were obtained and expressed as mean (range) or n (percentage).

### CPETs

All CPETs were performed in a similar method to those patients who underwent cardiopulmonary exercise testing enrolled in the US Melody valve investigational trial.^1^ CPETs were performed in the same lab, using the same equipment, and by the same exercise physiologist. A symptom-limited CPET progressive ramp protocol was performed on an electronically braked Corival CPET cycle ergometer (MGC Diagnostics, Saint Paul, MN). Participants were equipped either with a neoprene face mask or with a rubber silicone mouthpiece with a saliva trap connected to an Ultima CardiO2 (MGC Diagnostics) metabolic cart. The workload was then increased continuously with a slope chosen to achieve each subject’s predicted maximal work rate (W) after 10-12 minutes of cycling. Participants were encouraged to keep a constant pedaling rate of 60-80 rpm. The test began with 2 minutes of unloaded cycling.

Expired gases were measured at rest and throughout the exercise protocol. Metabolic measurements including oxygen consumption (V_O2_), carbon dioxide production (V_CO2_), and minute ventilation (V_E_) were obtained on a breath-by-breath basis. The O_2_ pulse (V_O2_/HR) was measured at peak V_O2_, which was defined as the highest V_O2_ achieved by the subject during the test. Values for V_O2_ and work rate were indexed to body weight and expressed as a percentage of predicted values for healthy age- and gender-matched subjects as reported in previous studies with a similar protocol.^6^ Submaximal parameters of ventilatory efficiency (V_E_/V_CO2_) and ventilatory anaerobic threshold (VAT) were also measured. V_E_/V_CO2_ was measured at VAT, which was measured by the V-slope method. The RER and HR were measured continuously. Baseline and exercise spirometry data were not obtained.

### Imaging

Multiplanar magnetic resonance imaging (MRI)/computed tomography angiography (CTA) was performed before Alterra placement data were collected. Right ventricle (RV) and left ventricle (LV) volumes and function were assessed and indexed to the body surface area. Echocardiograms were performed on Epiq 7 (Philips, Cambridge, MA), and data were obtained on the same day as CPETs. Echocardiography data regarding tricuspid valve regurgitation characterization and gradient, RVOT mean and peak gradient, and severity of regurgitation across the RVOT or pulmonary valve were collected.

### Statistical analysis

CPET data are expressed as mean ± standard deviation (median or percentage). Paired samples were analyzed using a paired Student’s *t*-test using Microsoft Excel 2016. Statistical significance was set to *P* values of <0.05. Individual patient changes in V_O2_ and V_E_/V_CO2_ pulse were graphically represented in separate figures.

## Results

### Demographic data

A total of 13 patients were identified. Four patients did not have a baseline CPET and 1 did not have a follow-up CPET. One patient was excluded who did not achieve the minimum RER and HR, leaving an eligible study cohort of 7 patients.

Of the 7 patients, 5 (71%) were male, and the mean age was 22 years (range: 12-49 years). The mean weight was 60.6 kg (range: 32.7-81.6 kg), with a mean body mass index of 21.3 (range: 14.5-32.7). Five patients (71%) had an original lesion of tetralogy of Fallot, 1 patient (14%) had double outlet RV with subaortic ventricular septal defect and pulmonary stenosis, and 1 patient (14%) had normal segmental anatomy with isolated pulmonary valve stenosis. The average number of previous surgeries was 1 (range: 0-4), with an average time from original repair to Alterra placement of 22 years (range: 13-48 years) (see Table 1).

**Table 1:** Demographic and MRI/CTA Data (n = 7)

Age (y)	22 (12-49)
Weight (kg)	60.6 (32.7-81.6)
BMI	21.3 (14.5-32.7)
Sex (male)	5 (71%)
Original lesion	
TOF	5 (71.4%)
DORV	1 (14.3%)
PV stenosis	1 (14.3%)
Number of surgeries	1 (0-4)
Time from original repair (y)	22.0 (12.6-48.1)
Prestent indication	
Regurgitation	7 (100%)
Cardiac MRI/CTA	
Right ventricular end-diastolic volume (mL/m^2^)	179 ± 35 (189)
Right ventricular end-systolic volume (mL/m^2^)	108 ± 34 (103)
Right ventricular stroke volume (mL/m^2^)	71 ± 27 (71)
Right ventricular ejection fraction (%)	41 ± 13 (41)
Pulmonary regurgitation fraction (%)	41 ± 13 (37)

Data are presented as mean (range), n (%), or mean ± SD (median).

BMI, body mass index; CTA, computed tomography angiography; DORV, double outlet right ventricle; MRI, magnetic resonance imaging; PV, pulmonary valve; SD, standard deviation; TOF, tetralogy of Fallot.

### CPET data

Baseline CPET data were obtained on average 1.9 ± 2.3 days before Alterra placement and 186.1 ± 3.4 days after placement. Pre- and post-Alterra placement CPET data showed improvement of V_E_/V_CO2_ (downward trend) at VAT in 6 of 7 (86%) patients with a statistically significant improvement in the mean V_E_/V_CO2_ (pre: 28.8 ± 4.2, post: 26.0 ± 3.0) (*P* = 0.03). The mean percent predicted peak V_O2_ (pre: 71.0% ± 14.7% predicted, post: 72.6% ± 9.9% predicted), mean percent predicted oxygen pulse (pre: 83.6% ± 14.1% predicted, post: 86.7% ± 13.9% predicted), and mean percent predicted peak work rate (pre: 69.6% ± 25.3% predicted, post: 74.1% ± 7.3% predicted) increased in number comparing pre- and post-Alterra CPET data but were not statistically significant (see Table 2).

**Table 2:** Pre- and post-Alterra CPET and echocardiography data (n = 7)

	Pre	Post	Difference	*P* value
Days from Alterra placement	−1.9 ± 2.3 (−1.0)	186.1 ± 3.4 (186.1)	188.0	
CPET				
Peak V_O2_ (mL/kg/min)	27.1 ± 9.8 (31.4)	29.0 ± 8.5 (29.0)	1.9 ± 8.9	0.5583
Peak V_O2_ (% predicted)	71.0 ± 14.7 (72.5)	72.6 ± 9.9 (72.6)	1.6 ± 12.0	0.7185
Oxygen pulse (% predicted)	83.6 ± 14.1 (85.9)	86.7 ± 13.9 (86.7)	3.2 ± 13.5	0.5112
Work rate (W/kg)	2.0 ± 0.9 (1.7)	2.3 ± 0.8 (29.0)	0.3 ± 0.8	0.3964
Work rate (% predicted)	69.6 ± 25.3 (76.8)	74.1 ± 7.3 (74.1)	4.5 ± 18.1	0.5655
V_E_/V_CO2_ ratio at VAT	28.8 ± 4.2 (28.2)	26.0 ± 3.0 (26.0)	−2.8 ± 3.8	0.0334
RER	1.2 ± 0.1 (1.1)	1.2 ± 0.1 (1.2)	0.0 ± 0.1	0.9546
Peak HR (beat/min)	167.7 ± 20.1 (178.0)	166.4 ± 14.7 (166.4)	−1.3 ± 16.9	0.8844
Echocardiography				
Tricuspid regurgitation (TR), n (%)				
None/mild	7 (100)	7 (100)		
TR peak gradient (mm Hg)	26.8 ± 6.4 (26.0)	31.3 ± 15.3 (29.0)	4.5 ± 11.8	0.1941
RVOT peak (mm Hg)	13.3 ± 3.0 (14.0)	16.0 ± 5.7 (14.0)	2.7 ± 4.6	0.3201
RVOT mean (mm Hg)	7.4 ± 2.4 (7.0)	9.0 ± 3.4 (8.0)	1.6 ± 2.9	0.3764
Pulmonary valve regurgitation, n (%)				
None/trivial		6 (86)		
Mild/moderate		1 (14)		
Severe+	7 (100)			

Data are presented as mean ± SD (median) unless otherwise specified.

CPET, cardiopulmonary exercise test; HR, heart rate; RER, respiratory exchange ratio; RVOT, right ventricular outflow tract; SD, standard deviation; VAT ventilatory anaerobic threshold.

**Figure 1:**
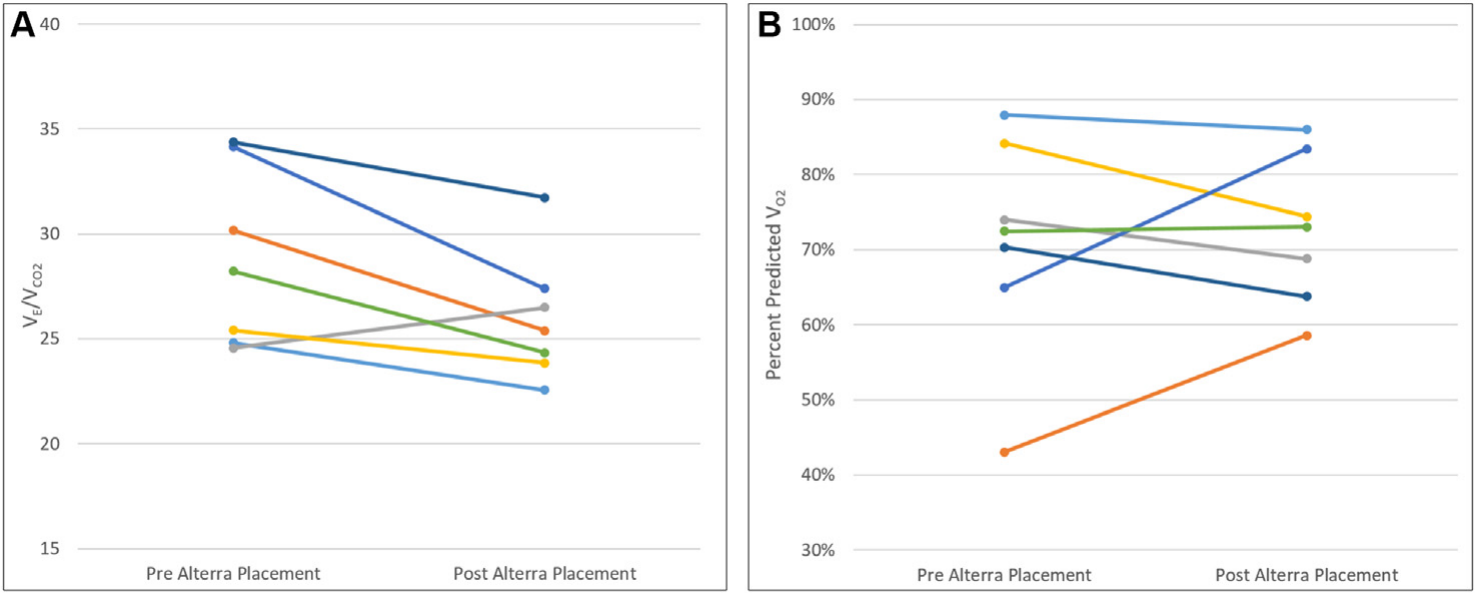
(**A**) Change in V_E_/V_CO2_ over time from pre- and post-Alterra placement. (**B**) Change in percent predicted V_O2_ over time from pre- and post-Alterra placement.

Changes in the percent predicted V_E_/V_CO2_ and V_O2_ for each patient were separately graphed using simple line charts to better visualize which patients had a preferential change to allow further assessment. Six of the 7 (86%) patients had improvement in V_E_/V_CO2_ (see Fig. 1A). Two of the 7 (29%) patients had clinically significant improvement of peak predicted V_O2_, who were the 2 individuals with the lowest starting value (see Fig. 1B).

### Imaging data

Before Alterra placement, cardiac MRI/CTA data were obtained (see Table 1). Echocardiography data before and after Alterra placement were obtained; all showed improvement in pulmonary valve/RVOT regurgitation with no other statistically or clinically significant changes (see Table 2).

## Discussion

This case series provides early data regarding exercise function among patients with Alterra prestent and SAPIEN 3 THV placement. Although the size of the cohort prevents strong conclusions to be made, there were 2 key observational trends of interest. Patients in our cohort who presented for the Alterra placement appear to have a more dilated RVOT and higher right ventricular end-diastolic volume (RVEDV) compared with those with placement of the Melody or SAPIEN 3 valve^7^ and V_E_/V_CO2_ decreased, demonstrating improvement in most patients, which is different from previous studies in which subanalysis was performed on patients who had primarily pulmonary regurgitation.^8^

The patients in our cohort who presented for the Alterra appear to have a more dilated RVOT and higher RVEDV by MRI compared with those who received the traditional Melody or SAPIEN 3 valve.^7^ Pre-PPVI rest MRIs in the Lurz et al.^7^ study showed an RVEDV (mL/m^2^) of 113.8 ± 41.0 compared with our cohort that showed an RVEDV (mL/m^2^) of 179 ± 35 (see Table 1). For reference, a normal RVEDV would be approximately 94 ± 15 mL/m^2^ in men and 78 ± 12 mL/m^2^ in women with a mean age of 20-29.^9^ Although there was a difference in RVEDV, the baseline peak predicted V_O2_ percentage of our cohort (71.0 ± 14.7) was similar to the pulmonary regurgitation cohort (66 ± 17) previously studied by Lurz et al.^8^

A comparison of our cohort specifically with the pulmonary regurgitation cohort was chosen given that 100% of our cohort had severe+ pulmonary regurgitation on echocardiogram before Alterra placement (see Table 2). Neither of these groups demonstrated a significant increase in peak predicted V_O2_ after PPVI, and therefore, one may extrapolate that RV dilation alone is not a good indicator to predict improvement in exercise capacity after PPVI. Furthermore, our data do not suggest that patients would benefit from earlier intervention, but rather, patients who already have low peak predicted V_O2_ will likely have the greatest improvement (see Fig. 1B). This emphasizes the value of CPETs among patients reporting exercise intolerance or patients who may have borderline indications for PPVI. It also raises the question of whether an isolated intervention such as PPVI would be enough to improve exercise capacity alone. Presumably, patients, such as our cohort, who in general do not have myocardial insufficiencies like patients with ischemia or cardiomyopathies may also benefit from concomitant enrollment in a cardiac or fitness rehabilitation programme.

Interestingly, our cohort did demonstrate an improvement in the mean ventilatory efficiency (V_E_/V_CO2_), represented by a decrease in number, along with individual improvement in all but one patient. This is in contrast to prior studies that have shown that patients with predominant pulmonary regurgitation do not show statistical improvement.^8^ Traditionally, V_E_/V_CO2_ improvement has been isolated to patients after PPVI who had predominant pulmonary stenosis.^8^ In general, elevations of V_E_/V_CO2_ may be seen for multiple reasons, for example, in patients with impaired transport of gases across the alveolar-capillary membrane (eg, patients with elevated pulmonary capillary wedge pressures), right to left intracardiac shunts, or pulmonary vascular disease. It can also be seen in patients secondary to the absence of a subpulmonary ventricle and suboptimal distribution of pulmonary blood flow resulting in ventilation/perfusion mismatch.^10^ Therefore, it makes physiological sense that patients with pulmonary stenosis who undergo PPVI would have normalization of pulsatile blood flow and improvement in V_E_/V_CO2_. However, it brings to question why our cohort of patients with predominant pulmonary regurgitation showed a significant change. Perhaps patients who have larger RV volumes, such as our cohort, may have more right ventricular strain that has been shown on echocardiograms to correlate with improvement of V_E_/V_CO2_ after PPVI.^11^ It could also be because after Alterra placement there was improved pulmonary blood flow misdistribution decreasing ventilation/distribution mismatching, which was also speculated in the Melody valve trials.^1^ To better assess and validate this finding, future studies could perform post-Alterra placement cardiac MRIs to characterize RV function and strain. Studies could also assess for any correlation between the amount of baseline pulmonary valve regurgitation percentages and changes in V_E_/V_CO2_.

The findings from this study provide some early insight into changes in exercise capacity among patients receiving percutaneous Alterra placement compared with published data in other percutaneous pulmonary valve systems. This study also highlights the value of CPET testing when evaluating or even monitoring patients who may require pulmonary valve replacement. Future studies can add to the clinical decision-making and may help by better defining what specifically “exercise intolerance” means for patients undergoing evaluation for pulmonary valve replacement criteria.^12^

This study was limited by power and the relatively short amount of time after Alterra implantation, making it difficult to predict long-term changes or improvement. The small cohort of patients makes it difficult to draw definitive conclusions. This retrospective study is also missing important data such as repeat cardiac MRI/CTA that may help guide understanding RV function and volume changes after PPVI.

## Conclusion

Early exercise function data looking at patients before and after pulmonary valve implantation with the Alterra Adaptive Prestent and SAPIEN 3 THV system implantation show that patients had a more consistent improvement in ventilatory efficiency (V_E_/V_CO2_) compared with other CPET parameters. This may be suggestive of favourable haemodynamic changes conceivably from improvement in right ventricular strain although further investigation is required with larger patient volumes and longer follow-up.
